# A pilot study to prevent a thin endometrium in patients undergoing clomiphene citrate treatment

**DOI:** 10.1186/1757-2215-6-94

**Published:** 2013-12-27

**Authors:** Akihisa Takasaki, Hiroshi Tamura, Toshiaki Taketani, Katsunori Shimamura, Hitoshi Morioka, Norihiro Sugino

**Affiliations:** 1 Department of Obstetrics and Gynecology, Saiseikai Shimonoseki General Hospital, Yasuokacho 8-5-1, Shimonoseki 759-6603, Japan; 2 Department of Obstetrics and Gynecology, Yamaguchi University Graduate School of Medicine, Minamikogushi 1-1-1, Ube 755-8505, Japan

**Keywords:** Clomiphene citrate, Thin endometrium, Antiestrogen

## Abstract

**Abstracts:**

**Background**

Clomiphene citrate (CC) is most commonly used as a first-line treatment of infertility. However, a disturbance of endometrial growth by the adverse effects of the CC has been recognized. Since a thin endometrium is recognized as a critical factor of implantation failure, preventing CC-induced thinning of the endometrium is important. This study was undertaken to investigate whether the modified CC treatments are useful to prevent a thin endometrium in patients undergoing CC treatments.

**Methods**

This study is a prospective, randomized controlled study. The study was performed at the Saiseikai Shimonoseki General Hospital during a 4-month period (May 2012 to September 2012). Sixty-six infertile women who had a thin endometrium (< 8 mm) during the standard CC treatment (50 mg/day on days 5–9 of the menstrual cycle) were enrolled. The patients were randomly divided into three groups: 22 patients were given 25 mg/day CC on days 5–9 (half-dose group), 22 patients were given 50 mg/day CC on days 1–5 (early administration group) and 22 patients received a standard CC treatment again (control group). Endometrial thickness at the induction of ovulation was assessed by ultrasonography. The primary endpoint of this study was an endometrial thickness.

**Results**

Half dose administration and early administration improved the endometrial thickness (≥ 8 mm) in 14 patients (70%) and in 19 patients (90%) respectively, while only 3 patients (15%) improved in endometrial thickness in the control group. The mean endometrial thickness was also significantly higher in the half dose group (8.6 ± 1.5 mm) and early administration group (9.4 ± 1.5 mm) compared to the control group (6.7 ± 1.8 mm). No side effect was observed in this study.

**Conclusions**

The modified treatment with a half-dose or early administration of CC significantly increased endometrial thickness in patients with a history of thin endometrium caused by the standard CC regimen. The modified CC treatments in this study can be beneficial for patients with a thin endometrium as a result of standard CC treatment.

**Trial registration**

Clinical Trial Registration Number: UMIN000007959.

## Background

Clomiphene citrate (CC) has been widely used to stimulate follicular growth in the treatment of infertility. CC has an antiestrogenic effect on the hypothalamus by binding to estrogen receptors. This stimulates a gonadotropin-releasing hormone (GnRH) pulse that induces gonadotropin secretion from the anterior pituitary gland. CC is most commonly used as a first-line treatment of infertility [1,2]. However, several adverse effects of the CC treatment have been recognized. One of them is a disturbance of endometrial growth by the antiestrogenic effect. In fact, endometrial thickness is significantly thinner in women taking CC than women not taking CC [3-5]. Since a thin endometrium is recognized as a critical factor of implantation failure [6-9], preventing CC-induced thinning of the endometrium is important.

Since the endometrium grows by estrogen during the follicular phase, endometrial growth is impaired by antiestrogenic effects of CC [10]. Impaired epithelial cell proliferation and delayed glandular maturation are often found in endometrial biopsies from patients treated with CC [11]. Some reports also indicate that the number and diameter of the gland were lower in the CC treatment cycle than in the control cycle [12]. These results strongly suggest that a thin endometrium caused by the CC treatment is due to impaired endometrial growth and that this is a major cause of poor pregnancy rates in women who showed a thin endometrium by the CC treatment. Therefore, these patients receive the next step therapy such as gonadotropin therapy, which potentially causes multiple pregnancies or ovarian hyperstimulation syndrome (OHSS).

To reduce the adverse effect of CC on endometrial growth, some clinical trials have been tested so far. Unfer et al. reported that ethinyl estradiol treatment reversed the antiestrogenic effect of CC on the endometrium [13]. High dose of phytoestrogens [14] and transdermal estradiol [15] were also tested in clinical trials to improve endometrial thickness in patients undergoing CC treatment. However, there are no established treatments to reduce or to reverse the adverse effect of CC on the endometrium.

In this study, we focused on the dosage and timing of CC treatment. Reduced amounts of CC (half dose) may diminish the antiestrogenic effect of CC because the antiestrogenic effect of CC is dose-dependent [16]. Furthermore, since the antiestrogenic effect of standard CC treatment continues until the late follicular phase when endometrial growth is still active, early administration of CC may reduce the adverse effect of CC on the endometrium. Therefore, this study was undertaken to investigate whether the modified CC treatments are useful to prevent a thin endometrium in patients undergoing CC treatments.

## Methods

This study (UMIN000007959) is a prospective, randomized controlled study. The study was conducted according to guidelines as stated in the Declaration of Helsinki and the protocol was approved by the Institutional Review Board of Saiseikai Shimonoseki General Hospital. Informed consent was obtained from all the patients. Patients were randomized at the beginning of each cycle by sealed opaque envelopes containing random generated numbers. The study was performed at the Saiseikai Shimonoseki General Hospital during a 4-month period (May 2012 to September 2012).

### Clinical studies

Women with a history of infertility, aged 20–42 yr, first received a standard CC treatment, 50 mg of CC (Clomid; Shionogi Co. Ltd., Tokyo, Japan) daily for 5 days starting on the 5th day of the menstrual cycle. Endometrial thickness and follicular growth were assessed by vaginal ultrasonography [Aloka ProSound SSD-3500SV type of instrument and Aloka UST-984-5 (5.0 MHz) vaginal transducer, Aloka Co. Ltd., Tokyo, Japan]. When follicles reached 18 mm or more in diameter by ultrasonography, 10,000 IU human chorionic gonadotropin (HCG, Gonatropin; Asuka Pharmaceutical Co. Ltd., Tokyo, Japan) was administered to induce ovulation. After a longitudinal view of the uterus was obtained, the thickness of the endometrium was measured at the maximum distance between each myometrial/endometrial interface on the day of HCG injection. When endometrial thickness was less than 8 mm, the patient was diagnosed as having a thin endometrium based on our previous studies [17,18]. The profiles of the patients in this study including the cause of infertility are shown in Table 1. There were no difference in the age and causes of infertility among the three groups. The patients were non-smokers and free from major medical illness including hypertension; patients were excluded if they had myoma, adenomyosis, congenital uterine anomaly, or ovarian tumors. Patients were also excluded if they used estrogens, progesterone, androgens, or had chronic use of any medication, including nonsteroidal anti-inflammatory agents. All patients showed no abnormal findings in the uterine cavity by hysterosalpingography (HSG).

**Table 1:** Profiles of the patients in this study

	**Control (N = 20)**	**Half dose (N = 20)**	**Early administration (N = 21)**
Age of women (years)	33.6 ± 4.1	33.3 ± 4.8	33.0 ± 4.8
Causes of infertitlity			
Ovulation	8	8	7
PCOS	1	1	2
Male factor	0	1	0
Unexplained	11	10	12
Endometrial thickness during the standard CC treatment cycle (mm)	6.8 ± 0.9	6.4 ± 0.8	6.3 ± 1.1

Data are shown as the mean ± SD.

Sixty-six patients who were diagnosed as having a thin endometrium (< 8 mm) during the standard CC treatment cycle were recruited in this study. They all showed normal endometrial thickness (≥ 8 mm) during spontaneous cycles. To prevent a thin endometrium in the next cycle, the 66 patients were randomly divided into three groups: 22 patients were given 25 mg/day CC on days 5–9 of the menstrual cycle (half-dose group), 22 patients were given 50 mg/day CC on days 1–5 of the menstrual cycle (early administration group) and 22 patients received a standard CC treatment again (control group). When follicles reached 18 mm or more in diameter by ultrasonography, HCG (10,000 IU) was administered to induce ovulation. Endometrial thickness and the number of follicles on the day of HCG injection were also assessed by ultrasonography. The primary endpoint of this study was an endometrial thickness. Venous blood was obtained during the mid-luteal phase (7 days after ovulation) for the determination of serum progesterone concentrations. Progesterone concentrations were measured by enzyme immunoassay (ST AIA-PACK PROG, Tosoh Co., Ltd., Japan). The minimal detectable concentration is estimated to be 0.1 ng/ml. Intra-assay and inter-assay coefficients of variation were 9.9% and 11.3%, respectively.

### Statistical analyses

Statistical analysis was carried out with SPSS for Windows 13.0. The Mann–Whitney U-test using the Bonferroni correction, Fisher’s test, and Kruskal Wallis H-test were employed as appropriate. Differences were considered to be significant if P < 0.05.

## Results

**Figure 1:**
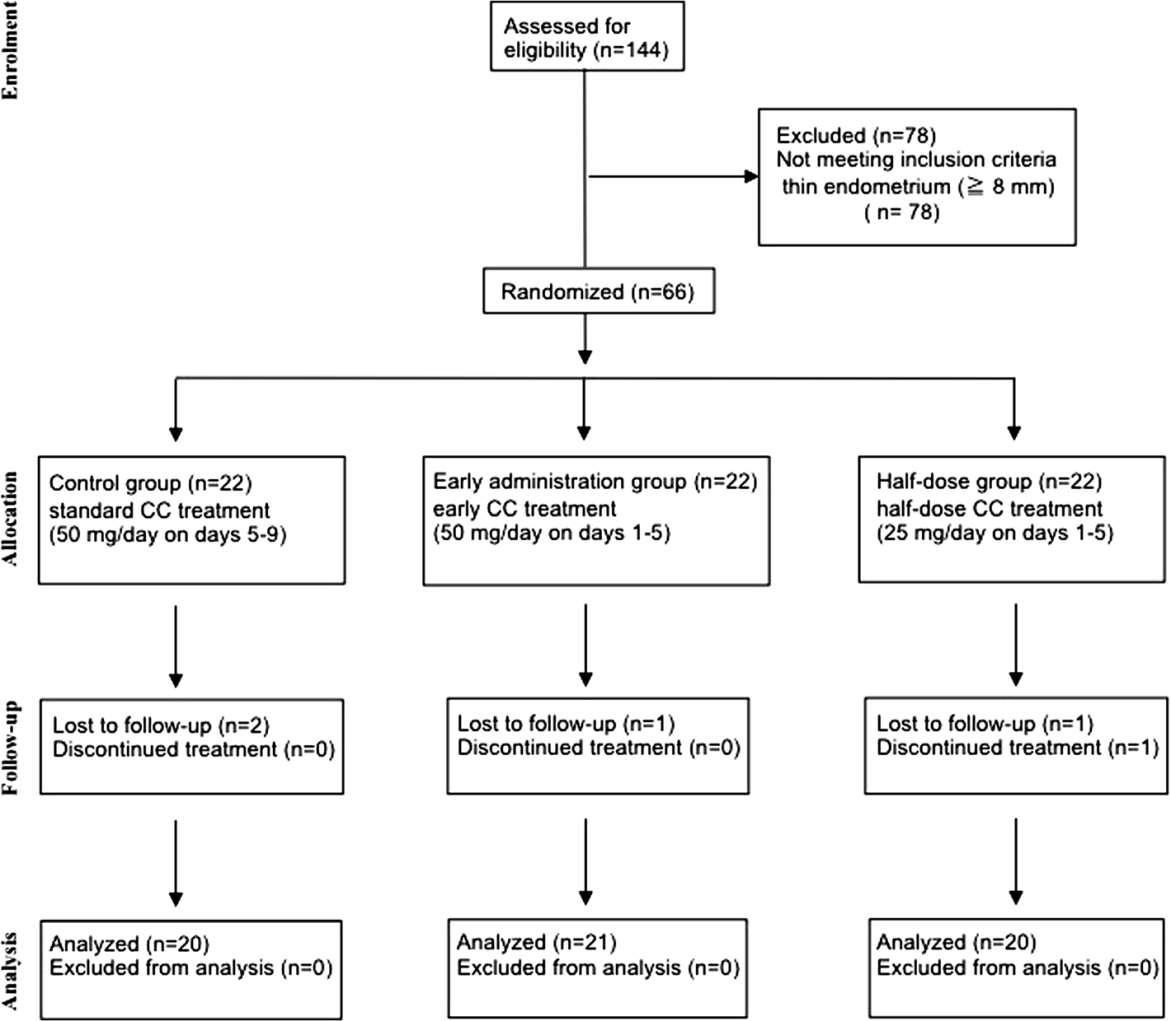
**CONSORT statement flow diagram.** In this RCT, sixty-six women who met the inclusion criteria were randomly divided to the three groups; control group (n-22), early administration group (n = 22), and half-dose group (n = 22).

The ages of women in the control, half-dose and early administration groups (33.6 ± 4.8, 33.8 ± 4.8 and 33.0 ± 4.8, respectively) were not significantly different (Table 1). None the infertility factors including ovulation disorder, PCOS, male factors, and unexplained infertility were significantly different among the three groups (Table 1). Since four patients were lost to follow-up and one patient discontinued the study due to anovulation, sixty-one participants were analyzed (control group: n = 20; early administration group: n = 21; half-dose group: n = 20) (Figure 1).

Endometrial thickness improved (≥ 8 mm) in 14 patients (70%) in the half-dose group, 19 patients (90%) in the early administration group and only 3 patients (15%) in the control group (Table 2). The mean endometrial thickness was also significantly improved in the half-dose group (8.6 ± 1.5 mm) and early administration group (9.4 ± 1.5 mm) compared to the control group (6.7 ± 1.8 mm) (Table 2). These results indicate that endometrial growth was significantly improved by the modified CC treatments.

**Table 2:** Endometrial thickness, number of follicles and serum progesterone levels in the control, half dose and early administration groups

	**Control (N = 20)**	**Half dose (N = 20)**	**Early administration (N = 21)**
Endometrial thickness			
Mean ± SD (mm)	6.7 ± 1.8	8.6 ± 1.5^a^	9.4 ± 1.5^a^
< 8 mm	17 (85.0%)	6(30.0%)	2(9.6%)
≥ 8 mm	3(15.0%)	14(70.0%)^a^	19(90.4%)^a^
Days until follicles maturation	12.0 ± 1.5	13.6 ± 2.7^b^	11.6 ± 2.3
Number of follicles			
≥ 15 mm	1.9 ± 1.0	1.8 ± 0.9	2.3 ± 0.9
≥18 mm	1.3 ± 0.5	1.4 ± 0.6	1.4 ± 0.6
Serum progesterone (ng/ml)	22.3 ± 11.3	18.8 ± 7.3	18.8 ± 10.3

Sixty-one patients were diagnosed as having a thin endometrium (< 8 mm) during the standard clomiphene citrate (CC) treatment cycle. To prevent a thin endometrium in the next cycle, the 61 patients were randomly divided into three groups: 20 patients were given 25 mg/day CC on days 5–9 of the menstrual cycle (half-dose group), 21 patients were given 50 mg/day CC on days 1–5 of the menstrual cycle (early administration group) and 20 patients received a standard CC treatment again (control group). Endometrial thickness and number of follicles was determined by vaginal ultrasonography on the day of HCG injection for ovulation induction. Venous blood was obtained for the determination of the mean ± SD. ^a^p < 0.05 versus Control, ^b^p <0.05 versus Control and early administration group. (Fisher’s test or Kruskal Wallis H-test).

The days until follicle maturation (days until HCG injection) were significantly longer in the half-dose group compared with the other groups (Table 2). There was no significant difference in the number of growing (≥ 15 mm) and mature follicles (≥ 18 mm) among the three groups (Table 2). Serum progesterone levels during the mid-luteal phase did not differ among the three groups (Table 2). One case in the half dose group and 2 cases in the early administration group became pregnant during the study cycle.

## Discussion

We often see patients with a thin endometrium as a result of standard CC treatment, which is the most common adverse effect of CC treatment. In fact, our preliminary study showed that 41 out of 100 women had a thin endometrium during a standard CC treatment cycle. Since a thin endometrium is a critical factor of implantation failure, we have to go to the next-step therapy such as gonadotropin therapy. Gonadotropin therapy not only burdens the patient with stress and medical expense but also can cause multiple pregnancy and OHSS. Therefore, preventing CC-induced thinning of the endometrium is important.

Our results showed that 14 out of 20 patients (70%) improved in endometrial thickness (≥ 8 mm) by half-dose of CC. This result seems to be inconsistent with a previous report by Dickey et al. [19] that endometrial thickness, determined on the day of HCG injection, was not related to the dose of CC (25–250 mg). This report was based on retrospective data of the endometrial thi]ckness from all women who underwent CC treatment while our study enrolled women whose endometrial thickness was thin (< 8 mm) in the previous CC treatment cycle.

One may raise a possibility that a half dose of CC influences follicular growth and luteal function. The half dose CC treatment delayed follicle maturation compared with a standard CC treatment. However, the difference was modest to be permissible. Furthermore, serum progesterone levels during the mid-luteal phase did not differ between the half dose group and the control group, suggesting that luteal function is not affected by the half dose CC treatment.

Our results also clearly showed that early administration of CC improved endometrial thickness in 19 out of 21 patients (90.4%). The effect of the timing of CC administration on endometrial thickness has been reported [20-22]. Early administration of CC (100 mg CC; day 1–5) showed higher endometrial thickness compared with the standard CC treatment (100 mg CC; day 5–9), but it was not significant [20-22]. These studies enrolled all the infertile women who showed and did not show a thin endometrium during the standard CC treatment. However, our study enrolled the women whose endometrial thickness was thin (< 8 mm) in the standard CC treatment cycle. Because early administration of CC stimulates follicular growth before dominant or subdominant follicles are selected, it might increase the number of growing follicles. However, our result showed that the number of growing follicles (≥ 15 mm) and mature follicles (≥ 18 mm) was not increased by early administration of CC.

Recently, the efficacy of letrozole, which is an aromatase inhibitor, as an ovulation inducing drug has been reported [23]. There is a possibility that letrozole can be used as an alternative ovulation inducing drug to prevent a thin endometrium. However, it is high cost, and further studies are needed on the safety of letrozole.

## Conclusions

The modified CC treatments provide an alternative to proceeding to gonadotropin therapy for patients with a thin endometrium as a result of standard CC treatment. The present study provides important information to prevent thin endometrium in patients undergoing CC treatment. However, our ultimate goal of CC treatment for infertile patients is a successful pregnancy and live birth. Large scale-RCT will be necessary to evaluate the efficacy of the modified CC treatment on successful pregnancy in patients with a history of a thin endometrium caused by the standard CC regimen.

## Competing interests

The authors declare that they have no competing interests.

## Authors’ contributions

AT designed the study and collected the data. HT analyzed the data and drafted the first manuscript. TT analyzed the data. KS and HM collected the data. NS directed the research and drafted the final manuscript. All authors approved the final manuscript.
